# Painful Abdominal Outpouching in an Adult Male

**DOI:** 10.7759/cureus.12416

**Published:** 2021-01-01

**Authors:** Angela G Zaladonis, Danielle Applebaum, Sylvia Hsu

**Affiliations:** 1 Dermatology, Temple University Hospital, Philadelphia, USA; 2 Dermatology, Elite Dermatology, Houston, USA

**Keywords:** post-herpetic, herpes zoster virus, pseudohernia, shingles

## Abstract

Post-herpetic abdominal pseudohernia is a neurologic complication of herpes zoster resulting from paresis of the abdominal wall muscles ipsilateral to the eruption. This poorly known condition may raise suspicion for true abdominal wall hernia or other concerning etiologies, resulting in extensive work-up and imaging. Post-herpetic abdominal pseudohernia is a relatively benign condition, which resolves spontaneously in the majority of cases. Therefore, it is important for the clinician to be aware of this complication in order to avoid unnecessary imaging or excessive management, which may increase the cost of care and burden to the patient.

## Introduction

Herpes zoster (HZ) is a clinical syndrome resulting from reactivation of the varicella-zoster virus (VZV). Initial infection with VZV generally occurs in childhood, resulting in varicella infection. Following resolution of the primary infection, VZV remains dormant in the dorsal root ganglia, autonomic ganglia, and cranial nerve ganglia. Host-specific immunity against VZV may wane with aging, stress, or state of immunosuppression, resulting in HZ, which is a spontaneous reactivation of the VZV. HZ is generally characterized by a vesicular rash distributed in a unilateral dermatomal fashion [1,2].

Segmental zoster paresis, or muscle zoster, is a neurologic complication of HZ, which results in a lower motor neuron weakness of the associated dermatome [1-3]. About 5% of all patients with HZ will develop some form of zoster paresis [3]. Post-herpetic abdominal pseudohernia (PHAP), first described in 1896, is the result of weakness to the abdominal wall by segmental zoster paresis of the abdominal muscles. It is found in about 2% of all patients diagnosed with HZ, although estimates range [2,4,5]. We describe the case of an adult male diagnosed with HZ who later developed an abdominal outpouching determined to be PHAP.

## Case presentation

**Figure 1:**
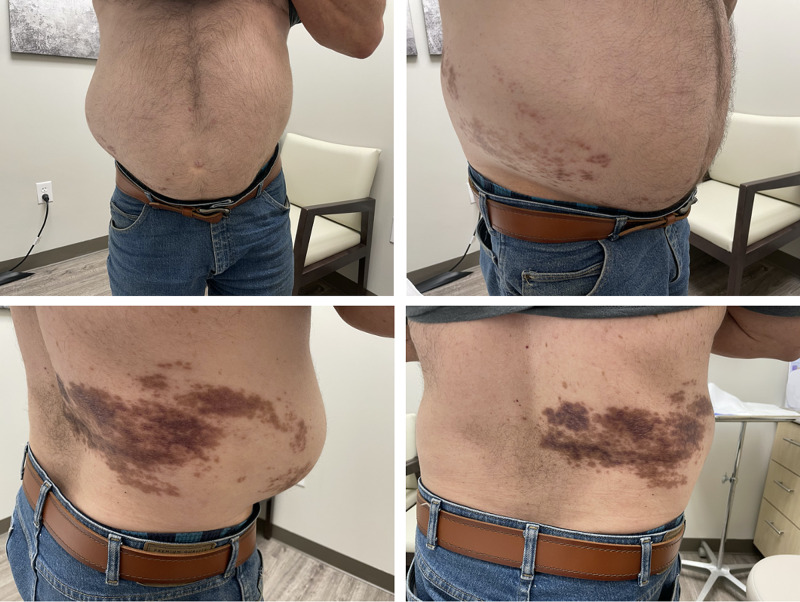
Post-Herpetic Abdominal Pseudohernia.

A 57-year-old man with history of diabetes mellitus and hypertension was diagnosed with HZ by his primary care provider. The patient was treated with valacyclovir. After one month, the initial dermatologic condition had resolved. However, the patient reported pain, skin hyperpigmentation, and an area of outpouching on the right abdomen and flank in the region of prior HZ eruption (Figure 1). His primary care provider ordered an ultrasound and computed tomography (CT) scan to look for ascites. Imaging studies were negative for ascites. The patient presented to our clinic for further evaluation. He was diagnosed with post-herpetic neuralgia and PHAP. The patient was reassured that the PHAP will likely resolve on its own and declined treatment with gabapentin for the neuralgia. On follow-up, he reported the pseudoherniation had already begun to reduce in size over the following month.

## Discussion

PHAP is a rare complication of HZ associated with segmental zoster paresis. Patients with this condition may exhibit bulging asymmetry of the abdomen, a reducible mass of the abdomen, or diminished or absent abdominal reflexes in the context of recent HZ [2-6].

PHAPs are more commonly found in the T10-T12 dermatome, in males, and those in their seventh decade. It usually presents within the first three to four weeks following HZ eruption but may present up to two months following diagnosis. On rare occasions the pseudohernia may present prior to eruption of HZ or in the absence of any HZ eruption [2].

Diagnosis of PHAP may be made clinically by assessment of presentation and history [2,5]. Some providers may consider using ultrasound, CT, or MRI to rule out other etiologies, including true abdominal hernia, tumor, or other visceral involvement. However, these are unnecessary and costly, unless the patient’s presentation is atypical or dubious. Additionally, some providers may consider electromyography to confirm diagnosis, but this should also be discouraged due to its invasive burden on the patient [6].

Unlike true abdominal hernias, PHAPs do not require surgery. In fact, up to 80% of all patients with PHAP will show spontaneous recovery by one year, with a median recovery time of about five months [2]. For the remaining patients who do not experience full recovery, conservative management with supportive garments, physical therapy, and analgesia offer symptomatic management.

## Conclusions

We report the uncommon case of an adult man presenting with a painful abdominal outpouching. Although rare, clinicians should consider the diagnosis of PHAP in a patient with this presentation because of its noninvasive work-up and benign disease course. Evaluation with history and physical exam are sufficient to make a clinical diagnosis of PHAP. Extensive imaging and testing are unnecessary, resulting in increased health-care costs and increased treatment burden on the patient.
